# Simultaneous monitoring of cerebral perfusion and cytochrome c oxidase by combining broadband near-infrared spectroscopy and diffuse correlation spectroscopy

**DOI:** 10.1364/BOE.9.002588

**Published:** 2018-05-10

**Authors:** Ajay Rajaram, Gemma Bale, Matthew Kewin, Laura B. Morrison, Ilias Tachtsidis, Keith St. Lawrence, Mamadou Diop

**Affiliations:** 1 Imaging Program, Lawson Health Research Institute, 268 Grosvenor St., London, ON, N6A 4V2, Canada; 2 Department of Medical Biophysics, Western University, 1151 Richmond St., London, ON, N6A 3K7, Canada; 3 Medical Physics & Biomedical Engineering, University College London, Gower St., Bloomsbury, London, WC1E 6BT, United Kingdom; 4 mdiop@uwo.ca

**Keywords:** (170.3660) Light propagation in tissues, (170.3880) Medical and biological imaging, (170.6510) Spectroscopy, tissue diagnostics, (170.3890) Medical optics instrumentation

## Abstract

Preterm infants born with very low birth weights are at a high risk of brain injury, in part because the premature brain is believed to be prone to periods of low cerebral blood flow (CBF). Tissue damage is likely to occur if reduction in CBF is sufficient to impair cerebral energy metabolism for extended periods. Therefore, a neuromonitoring method that can detect reductions in CBF, large enough to affect metabolism, could alert the neonatal intensive care team before injury occurs. In this report, we present the development of an optical system that combines diffuse correlation spectroscopy (DCS) for monitoring CBF and broadband near-infrared spectroscopy (B-NIRS) for monitoring the oxidation state of cytochrome c oxidase (oxCCO) – a key biomarker of oxidative metabolism. The hybrid instrument includes a multiplexing system to enable concomitant DCS and B-NIRS measurements while avoiding crosstalk between the two subsystems. The ability of the instrument to monitor dynamic changes in CBF and oxCCO was demonstrated in a piglet model of neonatal hypoxia-ischemia (HI). Experiments conducted in eight animals, including two controls, showed that oxCCO exhibited a delayed response to ischemia while CBF and tissue oxygenation (S_t_O_2_) responses were instantaneous. These findings suggest that simultaneous neuromonitoring of perfusion and metabolism could provide critical information regarding clinically significant hemodynamic events prior to the onset of brain injury.

## 1. Introduction

An estimated 15 million babies are born prematurely each year and those with very low birth weights (VLBW < 1500 g) are at a high risk of neurodevelopmental impairment [1]. Among the 63,000 VLBW infants born annually in the United States, 5-10% develop major disabilities such as cerebral palsy and 25-50% show other cognitive and behavioural deficits [2,3]. Although other factors contribute to perinatal brain injury, alterations in cerebral blood flow (CBF) are believed to play a significant role due to the immaturity of the cerebral vascular system and complications associated with premature birth, such as poor cardiac and lung function, which can impede blood flow and oxygenation [4]. In addition, changes in brain tissue metabolism are likely to precede structural abnormalities associated with injury [5]. In term infants, metabolite ratios measured by magnetic resonance (MR) spectroscopy have shown high accuracy in predicting adverse neurodevelopmental outcome [6,7] and have potential in guiding clinical management. Despite this, applications of MR methods for routine brain monitoring are clearly impractical. Cranial ultrasound remains the first-line option in the neonatal intensive care unit (NICU), particularly for monitoring intraventricular haemorrhage. However, ultrasound is used to detect brain damage that has already occurred, rather than detecting pathophysiological events that are indicative of oncoming injury. This speaks to the need for bedside methods capable of detecting CBF and metabolic fluctuations which could act as prognostic markers of brain injury.

Advancements in biomedical optics have provided cost-effective alternatives for monitoring the brain with the aim of detecting hemodynamic/metabolic events that may precede brain injury. In particular, near-infrared spectroscopy (NIRS) – a safe, quantitative, and portable technology – is widely used to obtain estimates of cerebral oxygen saturation (S_t_O_2_), which has been used as a surrogate marker of CBF in the clinic [8]. NIRS can also directly measure CBF, either by dynamic contrast-enhanced (DCE) methods [9,10], or by diffuse correlation spectroscopy (DCS). DCS has been extensively validated against other perfusion techniques and it has the advantage of providing continuous monitoring [11].

The combination of NIRS and DCS has the potential to further enhance brain monitoring by combining flow and oxygenation measurements to determine the cerebral metabolic rate of oxygen (CMRO_2_) [8,12–15], which is a more sensitive marker of tissue viability [16]. Consequently, monitoring CMRO_2_ could help identify clinically significant changes in CBF considering the fact that only flow reductions large enough to exhaust the compensatory increase in oxygen extraction will have an impact on energy metabolism [9]. However, a potential challenge with continuous monitoring of CMRO_2_ is that it requires the combination of a number of parameters: CBF, S_t_O_2_, and arterial oxygen saturation (S_a_O_2_) – which have proven challenging to measure during asphyxia [16]. Furthermore, CMRO_2_ computation relies on assuming a fixed arteriovenous blood volume ratio, which may vary under pathological conditions [17–19].

A direct measure of cerebral energy metabolism can be obtained by measuring changes in the oxidation state of cytochrome c oxidase (CCO) – a key element in oxidative metabolism – which is directly related to mitochondrial ATP production [20]. CCO is the terminal electron acceptor in the electron transport chain (ETC): the final stage of oxidative metabolism. A unique copper dimer (Copper A) in the enzyme has an absorption peak around 835 nm in its oxidized form (oxCCO), but not in its reduced state. A change in the redox state represents a change in oxidative cellular metabolism. To accurately resolve changes in oxCCO, many wavelengths (broadband) are required as the concentration of CCO is 10% of the *in-vivo* hemoglobin concentration. Previous studies have shown that broadband NIRS (B-NIRS) measured oxCCO changes are associated with acute changes in metabolism following hypoxia-ischemia (see Bale et al. for a detailed review [20]). As well, changes in oxCCO measured by B-NIRS showed strong correlation with measures of cellular metabolism from magnetic resonance spectroscopy [21] and microdialysis [22]. Simultaneous monitoring of perfusion and oxygen metabolism, through combining DCS and B-NIRS, could therefore provide a valuable tool for neuromonitoring in the NICU. This report presents the development a hybrid B-NIRS/DCS system for real-time monitoring of S_t_O_2_, CBF, and ΔoxCCO. The ability of the instrument to capture dynamic oxygenation, blood flow, and metabolic changes was demonstrated in an animal model of neonatal hypoxia-ischemia that has been shown to cause rapid reductions in cerebral blood flow and oxygenation [15].

## 2. Methods

### 2.1 Instrumentation

**Fig. 1:**
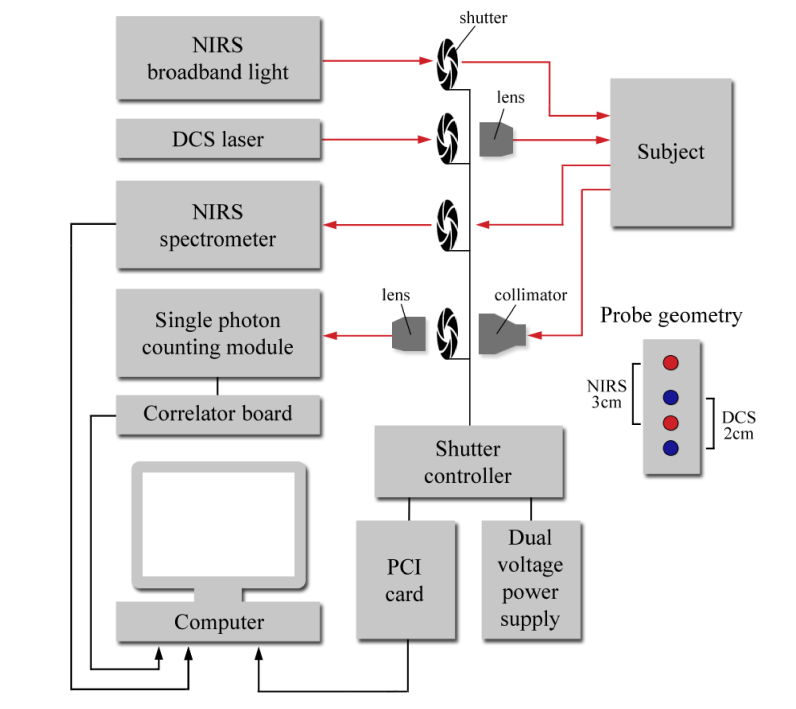
Simplified schematic of B-NIRS/DCS system with the shutter-based multiplexer. Red dots: B-NIRS emission and detection probes; blue dots: DCS emission and detection fibers.

The light source of the B-NIRS subsystem, a 20 W halogen lamp (Ocean Optics Hl-2000-HP), was high-pass filtered at 500 nm to remove ultraviolet light and directed towards the tissue by an optical fiber bundle (3.5 mm active diameter, 30-μm core, 0.55 numerical aperture). Light diffusely reflected from the tissue was collected with an identical fiber bundle, placed at 30 mm away from the emission probe (see Fig. 1

), and directed to a custom-made spectrometer (iDus Andor camera, Oxford Instruments; 548-1085 nm bandwidth; 1.65 nm resolution; P&P Optica, ON, Canada). The light source of the DCS was a long coherence length, continuous-wave laser emitting at 785 nm (DL785-100s, CrystaLaser, NV) and was coupled into a 400-μm diameter fiber. Light from the tissue was collected with a few-mode fiber (SMF-28e + , 8 µm core; few-mode at 785 nm) placed 20 mm from the DCS emission probe and coupled to a single-photon counting module (SPCM-AQR4C, Excelitas, QC, Canada). The output of the SPCM was fed into a correlator board (Flex033LQ-1, Correlator.com, NJ) to generate normalized intensity autocorrelation curves. Note that in some of the phantom experiments, a single-mode fiber (S630 Nufern, 6 µm core) was used instead of the few-mode fiber, as described in section 3.1.

Operating the two subsystems simultaneously would result in significant crosstalk since the DCS laser is powerful enough to saturate the spectrometer’s CCD detector. As well, the presence of incoherent light from the broadband light source alters the autocorrelation curves acquired while the B-NIRS light is on. To avoid crosstalk, a shutter-based multiplexing approach was used to alternatively collect measurements from each subsystem (i.e., B-NIRS and DCS). Figure 1 shows a schematic of the hybrid B-NIRS/DCS device.

Optical fibers were discontinuous on the NIRS emission and detection ends, with shutters placed at these junctions. Signal loss was minimized by securing the probes in close proximity to the shutter, using an optical breadboard. A shutter was also placed in front of the DCS laser, and another in front of the single-photon counting module. The latter was equipped with a light collimator and a coupling lens to maximize the signal-to-noise ratio (SNR). A dual DC power supply was used to operate the multiplexer: a high voltage (24 V) was sent to rapidly open the shutter, immediately followed by a lower voltage (10 V) to maintain the open state. These voltage values were chosen to minimize shutter blade transition time and digital modules (NI 9477, National Instruments, TX) were utilized to cycle between the voltage outputs. Opening and closing of the shutters were alternated to allow for sequential acquisition and were controlled using a field programmable gate array (FPGA) card (PCI-7811R, National Instruments). Digital modules (NI 9401, NI) were also used to control and monitor shutter states, which were synchronized by in-house software written in LabVIEW FPGA, as detailed in [23]. B-NIRS and DCS measurements were analysed using a customized MATLAB script (described in section 2.3) to yield cerebral oxygen saturation, perfusion, and metabolism.

### 2.2 System demonstration

#### 2.2.1 Phantom experiments

To investigate the potential impact of the B-NIRS light on the DCS measurements, autocorrelation curves were acquired (described in section 2.3.2) while the broadband light source remained on. Data were collected on a liquid phantom consisting of a mixture of Intralipid and India ink that provided a reduced scattering coefficient of 1.0 mm^−1^ and an absorption coefficient of 0.01 mm^−1^; values were chosen to mimic tissue based on previous reports [24]. In the first set of measurements, the emission probe of the B-NIRS was positioned 30 mm away from the detection fiber of the DCS system, and the level of light contamination experienced by the DCS was varied by modulating the intensity of the B-NIRS light using its built-in aperture. This test was repeated with a 10-mm distance between the B-NIRS emission probe and DCS detection fiber to further increase the intensity of B-NIRS light collected by the DCS system.

#### 2.2.2 Hypoxia-ischemia model

The ability of the hybrid B-NIRS/DCS device to monitor dynamic changes in CBF and oxCCO was demonstrated in an animal model of hypoxia-ischemia (HI) involving newborn piglets aged 10-40 hours. This model was chosen because it results in rapid changes in blood flow and oxygen supply to the brain [15]. Piglets were anesthetized under 3% isoflurane during preparatory surgery (2% post-surgery), tracheotomized, and mechanically ventilated on an oxygen-medical air mixture. Incisions were made lateral to the trachea and vascular occluders (In Vivo Metric, CA) were placed around the carotid arteries posterior to the clavicle. Catheters were inserted into an ear vein for injections and into a femoral artery to monitor vitals (SurgiVet, Smiths Medical, MN), as well as to collect arterial blood samples for gas and glucose analyses. Arterial oxygen saturation (S_a_O_2_) was measured via a pulse oximeter attached to the piglet’s right forelimb. Piglets were placed in the prone position and the B-NIRS and DCS probes were secured to the left side of the head, avoiding the sagittal sinus, using an in-house 3D-printed probe holder.

HI insult was induced by first inflating the occluders around the carotid arteries, followed by reducing the inspired oxygen from 21% to 8%. B-NIRS and DCS data were acquired continuously throughout the insult, starting 5 minutes prior to carotid clamping to acquire baseline measurements. The real-time DCS blood flow index was used to confirm successful clamping, i.e., an immediate drop in CBF. Following this confirmation, inspired oxygen concentration was reduced and the HI insult was maintained for a minimum duration of 10 minutes once the blood flow index reached its nadir. At the end of the HI insult, recovery was initiated by deflating the carotid occluders and returning oxygen supply to baseline levels. Two control animals experienced identical procedures, excluding inflation of the occluders and reduction in inspired oxygen, to confirm the stability of the optical measurements during the experiment.

The continuous recording of B-NIRS and DCS data were set on a 14-s cycle. That is, 12 B-NIRS spectra were acquired over 3 s, followed by 2 DCS measurements over the following 10 s, with a 0.5-s delay both preceding and following each technique. Data were continuously saved and imported into MATLAB for real-time update of the DCS blood flow index and relative oxCCO, as described in section 2.3. The final step in the experiment was to calibrate the DCS data [25] by measuring *absolute* CBF with DCE-NIRS following the 90 minute recovery (i.e. post HI insult) period. The DCE-NIRS protocol consisted of a bolus injection of indocyanine green (ICG, 0.1 mg/kg) into a cannulated vein. The passage of ICG through the brain was captured by continuously recording B-NIRS data at a temporal resolution of 400 ms. The time-varying arterial ICG concentration was concomitantly measured by a dye densitometer (DDG 2001, Nihon Kohden, Japan) attached to a front paw.

This study was approved by the Animal Use Subcommittee of the Canadian Council on Animal Care at Western University (London, Ontario).

### 2.3 Data processing

#### 2.3.1 Quantification of tissue chromophore concentrations and S_t_O_2_

##### 2.3.1.1 Broadband fitting for absolute concentrations

Before the start of each study, the spectrometer was wavelength calibrated using a neon light source and a dark-noise signal was also acquired. Thereafter, a reference spectrum was acquired – using a pinhole attenuator, which provided uniform attenuation while avoiding detector saturation – to account for the spectral properties of the light source and the spectrometer. A de-noising algorithm was subsequently applied to the data to reduce the measurement noise as described previously [26]. The baseline reflectance spectrum *R*(λ) was then computed from the spectrum measured on the piglet’s head, the dark-noise signal of the spectrometer, and the reference spectrum as follows:

$$R(\lambda )=\log_{10}\left(\frac{spectrum_{\lambda }-dark_{\lambda }}{reference_{\lambda }-dark_{\lambda }}\right)$$ (1)

The 1^st^ and 2^nd^ derivative of *R*(λ) was fitted to the 1^st^ and 2^nd^ derivatives of the solution to the diffusion approximation for a semi-infinite homogenous medium (i.e., the theoretical model) [10], characterized by the reduced scattering (*µ_s_’*) and absorption (*µ_a_*) coefficients, described respectively in Eqs. (2) and (3) [27]:

$$\mu_{s}^{'}=A{\left(\frac{\lambda }{800(nm)}\right)}^{-\alpha }$$ (2)

where *α* is the scattering power, characterizing the wavelength dependence of the *µ_s_’*, and *A* is the *µ_s_’* value at a wavelength λ = 800 nm (in our analysis, but can be any wavelength in the range where Eq. (2) is valid).

$$\mu_{a}(\lambda )=WF\cdot \varepsilon_{H_{2}O}(\lambda )+Hb^{b}\cdot \varepsilon_{Hb}(\lambda )+HbO_{2}^{b}\cdot \varepsilon_{HbO_{2}}(\lambda )$$ (3)

where WF is the tissue water fraction, *ε* is the extinction coefficient of the corresponding chromophore, and *Hb^b^* and *HbO_2_^b^* are respectively the baseline concentrations of deoxyhemoglobin and oxyhemoglobin in µM. CCO was not included in the baseline analysis due to its relatively small concentration and lack of features in the derivative spectra.

The baseline reflectance spectrum was analyzed using a three-step, multi-parameter fitting algorithm, based on a constrained least-square minimization algorithm built with a custom MATLAB function (*fminsearchbnd*) [10]. Firstly, the second-derivative of the reflectance spectrum *R*(λ) was fit to the 2^nd^ derivative of the theoretical model between 815 and 845 nm to obtain the tissue water fraction (*WF*). Although the fitting yielded estimates for all the parameters listed in Table 1

**Table 1:** Initial values, lower and upper bounds of the fitting parameters

Parameter	Initial value	Lower bound	Upper bound
*WF*	0.8	0.6	0.95
*Hb^b^(g/dL)*	10	0	60
*HbO_2_^b^(g/dL)*	40	0	80
*A* (mm^−1^)	1	0	1.3
α	2.7	0.6	4.0

, we only consider *WF* to be reliably estimated from this step because it has been previously shown that the 2^nd^ derivative of the scattering coefficient and the other tissue chromophores do not have significant contribution in this wavelength range [28]. Using the value of *WF* obtained in the first step as a known parameter, the 2nd derivative fit was performed from 680 to 800 nm to obtain *Hb^b^*. The *WF* and *Hb^b^* values were then used as known parameters in the fitting of the first derivative of *R*(λ) from 680 to 845 nm to determine *HbO_2_^b^*, A, and α [10,26,28]. The upper and lower bounds, as well as the initial values of the parameters used in the fitting routine are given in Table 1.

Baseline tissue oxygen saturation (*S_t_O_2_^b^*) was computed using *Hb^b^* and *HbO_2_^b^*:

$$S_{t}O_{2}^{b}=\frac{HbO_{2}^{b}}{Hb^{b}+HbO_{2}^{b}}$$ (4)

##### 2.3.1.2 Derivative spectroscopy for differential concentrations

Once the baseline S_t_O_2_ was quantified, a modified Beer-Lambert Law approach was used to analyze the complete set of B-NIRS spectra as it is considerably faster than the derivative fitting which takes 84 ± 17 s per spectrum on an i7-4700MQ 2.40 GHz processor. The UCLn algorithm was used to quantify changes in Hb, HbO_2_, and oxCCO concentrations (units: µM) from changes in attenuation across 770-900 nm [29], using a dynamic pathlength calculated by fitting the 2nd derivative of *R*(λ) to the second derivative of the water absorption spectra [28] and correcting for the wavelength dependence of the pathlength [30]. Tissue oxygen saturation at each time point was determined by combining the relative changes derived from the values obtained with the UCLn algorithm with the absolute baseline values obtained by the derivative fitting approach:

$$S_{t}O_{2}=\frac{\left(HbO_{2}^{b}+\Delta HbO_{2}\right)}{\left(Hb^{b}+\Delta Hb)+(HbO_{2}^{b}+\Delta HbO_{2}\right)}$$ (5)

where Δ*HbO_2_* and Δ*Hb* represent the relative changes in oxy/deoxy-hemoglobin.

#### 2.3.2 Quantifying CBF

DCS data were analyzed by fitting the measured electric field autocorrelation function to a solution to the diffusion approximation for a semi-infinite homogeneous medium [10]. Fitting was performed using the known source-detector distance (20 mm) and values of *µ_a_* and *µ_s_*′ at 785 nm obtained from the B-NIRS analysis. Time varying changes in *µ_a_* were used along with the baseline *µ_s_′* [25] to fit the dynamic data. The fitting of the autocorrelation curves yielded the correlation factor (β) and the blood flow index (BF_i_) [25].

For the DCE-NIRS analysis, CBF was quantified by relating the time-varying arterial concentration of ICG, C_a_(t), to the corresponding brain concentration curve, C_b_(t), using the following equation [31]:

$$C_{b}(t)=CBF\cdot R(t)*C_{a}(t)$$ (6)

where *R*(*t*) is the impulse residue function and * refers to the convolution operation. The product CBF • *R*(*t*) was extracted using a deconvolution algorithm [32], from which CBF was determined by the initial value since *R*(*t*) by definition begins at a value of one. The CBF measurement was then used to convert the DCS BF_i_ time series into physiological blood flow units (i.e., ml/100g/min) [25].

### 2.4 Error analysis

Monte Carlo simulations were conducted to assess the accuracy of the 3-step, multi-parameter fitting approach outlined in section 2.3.1.1. Simulated spectra were generated using the semi-infinite solution to the diffusion approximation with values of *Hb^b^* and *HbO_2_^b^* that corresponded to an S_t_O_2_ = 75.5% (all input parameters are given in Table 2

**Table 2:** Monte Carlo simulation of B-NIRS fitting algorithm

Simulation parameter	Input Values	1 acquisition	512 averages
*WF*	0.8	0.71 ± 0.13	0.80 ± 0.04
*Hb^b^(g/dL)*	13	12.0 ± 3.3	12.9 ± 0.6
*HbO_2_^b^(g/dL)*	40	36.2 ± 9.4	39.8 ± 1.7
*A* (mm^−1^)	0.8	0.93 ± 0.23	0.81 ± 0.04
α	2.6	2.6 ± 0.3	2.6 ± 0.03
S_t_O_2_ (%)	75.5	75.0 ± 3.9	75.5 ± 0.2

). Poisson noise was added at each wavelength, and the fitting routine used to obtain best-fit estimates of the 5 parameters. The same initial values and boundary conditions given in Table 1 were used. Simulations were run 500 times to generate a distribution of estimates for each fitting parameter. As well, the entire analysis was repeated by averaging spectra over 8 to 512 repetitions to estimate the precision of the fitting parameters over a range of SNR.

### 2.5 Statistical analysis

For the tissue phantom experiments, the influence of the NIRS broadband source on the estimated diffusion coefficients was tested using a Student’s t-test.

For the HI experiments, individual CBF time series, from each animal, were normalized to their corresponding baseline values. Thereafter, ten CBF values were extracted by dividing the normalized time series into bins of successive 10% reductions in flow and averaging within these bins. The corresponding oxCCO concentration changes were obtained for each CBF interval. A multivariate ANOVA was conducted to determine which oxCCO values were significantly different from baseline. The same approach was also applied to investigate the relationship between CBF and S_t_O_2_ during HI.

## 3. Results

### 3.1 Instrumentation

**Fig. 2:**
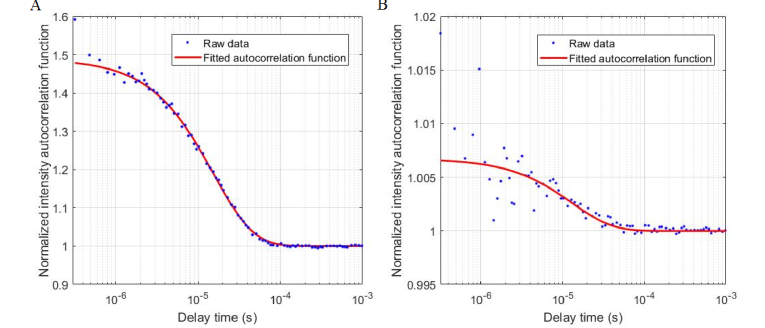
Autocorrelation functions (blue symbols) acquired with (a) DCS alone, and (b) DCS in the presence of light from the B-NIRS source (10 mm). The red lines in both figures are the best fit to the diffusion approximation.

The experiments conducted with the tissue-mimicking phantoms showed a significant effect of the broadband light source on the autocorrelation curves. Figure 2

presents curves acquired with the B-NIRS emission probe at 10 mm away from the DCS detection, along with the corresponding best fit to the diffusion approximation. Note that a single-mode fiber was used in this test to provide a wide dynamic range for the correlation factor, β. The curves shown in Fig. 2 were acquired with the B-NIRS light off (A) or on (B).

**Fig. 3:**
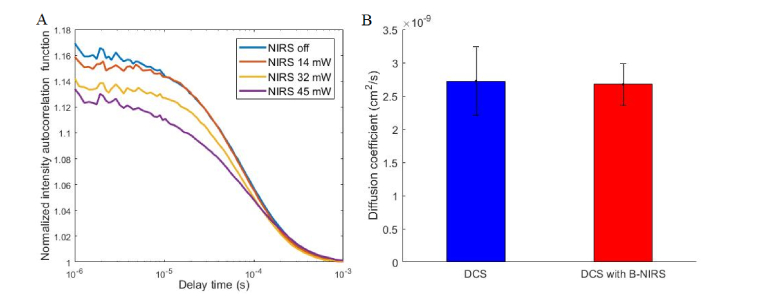
(A) DCS autocorrelation curves with B-NIRS light (30 mm from DCS detection) at varying intensities, (B) diffusion coefficient values without (blue) and with (red) the B-NIRS light; data were averaged over 10 acquisitions, error bars represent standard deviation.

As expected at this short distance between the B-NIRS emission and DCS detection, the high intensity of incoherent light from the broadband source significantly reduced the SNR of the acquired autocorrelation functions and the correlation factor β (y-intercept). The use of a few-mode fiber in this test would produce even lower β values, by allowing the propagation of more modes of light. A significant difference in the derived diffusion coefficient was found between the two curves: 1.56 ± 0.05 × 10^−8^ cm^2^/s (DCS alone) and 1.82 ± 0.30 × 10^−8^ cm^2^/s (with broadband light source on) (p < 0.01). The broadband light source also affected the estimated β value when using a few-mode fiber with a distance of 30 mm as shown in Fig. 3(A)

. However, in contrast to the data acquired at 10 mm, the broadband light contamination did not significantly affect the values of the derived diffusion coefficient when the B-NIRS emission probe was 30 mm away from DCS detection (Fig. 3(B)).

To demonstrate the robustness of the B-NIRS multi-parameter fitting, the algorithm described in section 2.3.1.1 was used to estimate the 5 fitting parameters from spectra of varying SNR (obtained by averaging a varying number of spectra). The parameters obtained by fitting a low SNR spectrum (obtained from one simulated spectrum with added Poisson noise, as described above) and one high SNR spectrum (obtained by averaging 512 spectra low SNR spectra) are displayed in Table 2, along with the resulting S_t_O_2_ value. Note that the mean and standard deviation displayed in Table 2 were obtained by repeating the simulations 500 times (i.e., Monte Carlo type approach).

**Fig. 4:**
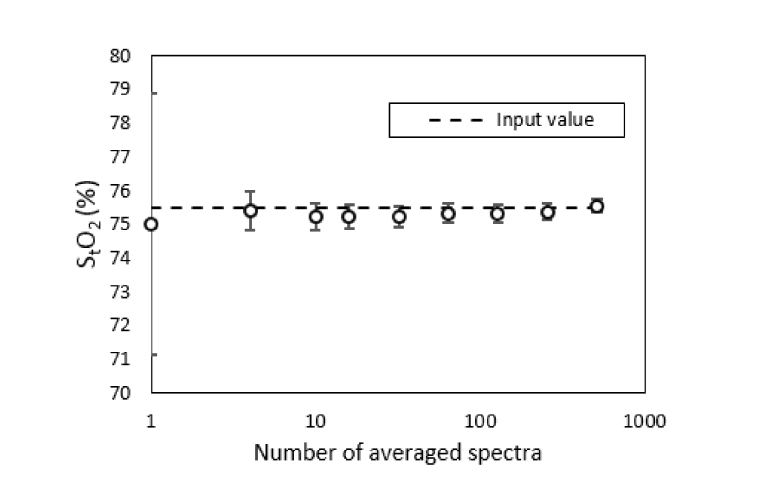
Estimation of S_t_O_2_ from simulated spectra of varying SNR (obtained by varying the number of averaged spectra). The circles represent the mean and the error bars are the standard deviation over 500 repetitions for each set of averaged spectra.

S_t_O_2_ values obtained by using a varying number of averaged spectra are displayed in Fig. 4

. The error bars represent the standard deviation over 500 simulations.

### 3.2 Hypoxia ischemia insult

Experiments were conducted on eight piglets (2 female, mean age = 25 ± 10 h, weight = 1.6 ± 0.3 kg). Of these, control experiments involving no HI insult were conducted on two animals. Table 3

**Table 3:** Baseline fitting parameters, S_t_O_2_, CBF

Parameter		Baseline value
*WF*	0.78 ± 0.02	
*Hb^b^(g/dL)*	18 ± 3	
*HbO_2_^b^(g/dL)*	35 ± 13	
*A* (mm^−1^)	0.35 ± 0.11	
α	3.3 ± 0.5	
S_t_O_2_ (%)	64 ± 13	
CBF (ml/100g/min)	28 ± 7	

displays the average baseline values across all animals for all 5 fitting parameters, cerebral oxygen saturation, and perfusion.

**Fig. 5:**
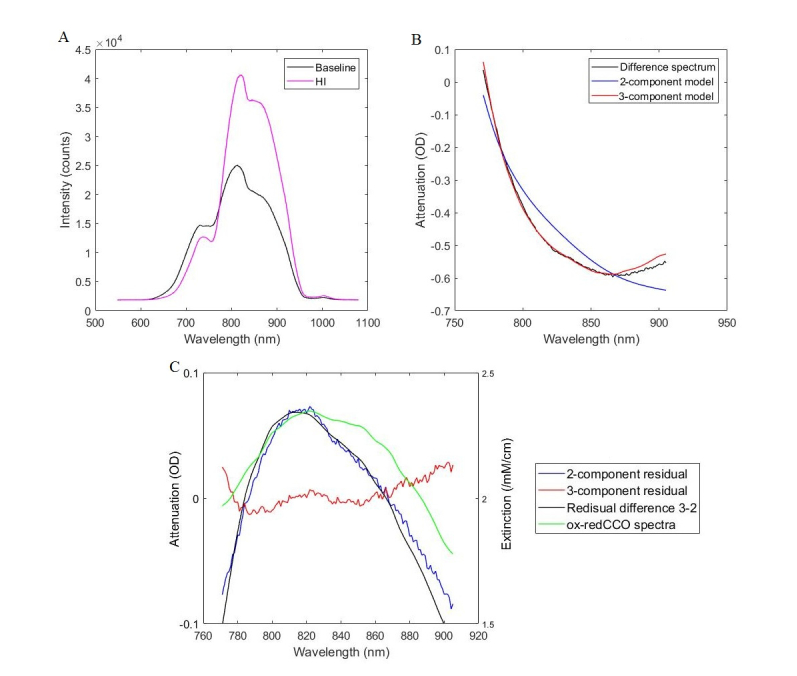
Broadband NIRS analysis showing (A) raw intensity measurements at baseline and during HI insult, (B) attenuation and best fit of the 2- (Hb and HbO_2_) and 3-component models (Hb, HbO_2_, and oxCCO), and (C) and residuals between measured and modelled attenuation, with CCO difference spectrum (ox-redCCO) for comparison.

B-NIRS spectra recorded at baseline and during the peak of HI insult are shown in Fig. 5(A)

. An attenuation difference, calculated as the log of the ratio between these spectra, is displayed in black in Fig. 5(B). The fit of the attenuation by the concentrations recovered from the Beer-Lambert algorithm are shown in blue and red. The blue curve represents the fit of HbO_2_ and Hb (2-component model), while the red line represents the fit of HbO_2_, Hb, and oxCCO (3-component model). It is clear that the addition of oxCCO to the model improved the fitting; this is further demonstrated in Fig. 5(C) which shows residuals between measured and modelled attenuation. The 3-component model has a smaller residual, randomly distributed around 0, whereas, the 2-component model had a residual centered on a peak around 820 nm. This suggests that there is a missing chromophore in the model with a peak at 820 nm, which is very similar to the oxCCO difference spectrum [20].

**Fig. 6:**
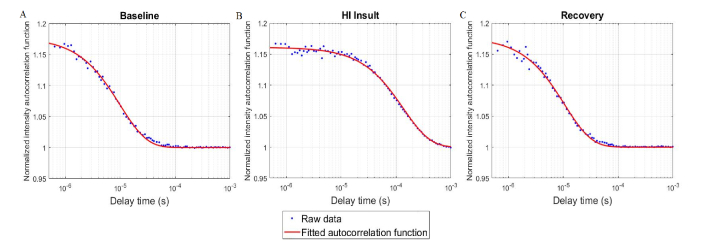
DCS autocorrelation curves measured in one piglet during (A) baseline (CBF = 35.2 ± 0.8 ml/100g/min), (B) HI insult (CBF = 2.6 ± 0.1 ml/100g/min), and (C) following insult recovery (CBF = 32.5 ± 0.8 ml/100g/min). The red lines represent best fit to the diffusion approximation.

A 5-s DCS acquisition period provided sufficient SNR with adequate temporal resolution to discern physiological changes caused by HI. Specifically, various DCS acquisition times were tested at baseline, and there was no statistically significant difference between the BF_i_ obtained at 5 and 15 s acquisitions over 30 cycles (difference = 3.2%, p = 0.42). DCS autocorrelation curves acquired at baseline, during HI insult, and following recovery are displayed in Fig. 6

. The CBF values derived by calibrating each BF_i_ were 35.2 ± 0.8 ml/100g/min, 2.6 ± 0.1 ml/100g/min, and 32.5 ± 0.8 ml/100g/min respectively.

Figure 7

**Fig. 7:**
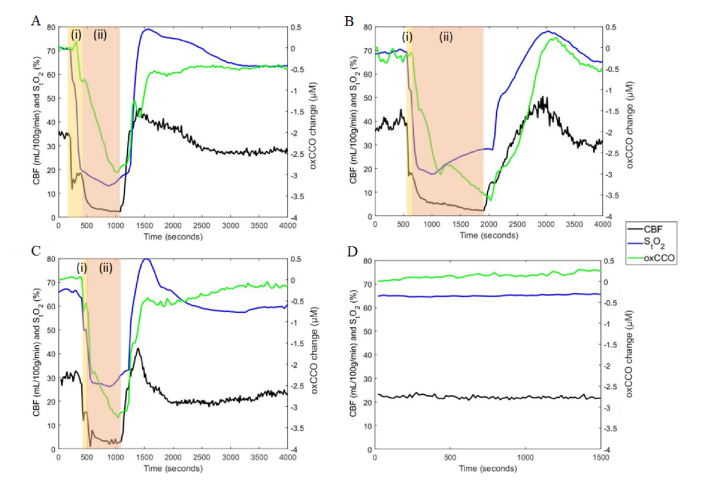
Simultaneous monitoring of S_t_O_2_, absolute CBF, and oxCCO in 3 hypoxia-ischemia (HI) animals (A, B, C) and a control piglet (D). The HI insult began with clamping the carotid arteries (region (i)), followed by inducing hypoxia by reducing the inspired oxygen fraction to 8% (region (ii)).

presents S_t_O_2_, CBF, and oxCCO time courses for three HI piglets and one control. Region (i) in each graph corresponds to the brief period between inflating the carotid occluders and reducing the inspired oxygen content. The combination of both components of HI is represented by region (ii). For the controls, no significant changes in the any of the parameters were detected. While S_t_O_2_ and CBF responded immediately to occluder inflation, oxCCO displayed a delayed response. As well, a continual reduction in oxCCO persisted after CBF and S_t_O_2_ had reached their nadir. This pattern was observable in all HI experiments.

**Fig. 8:**
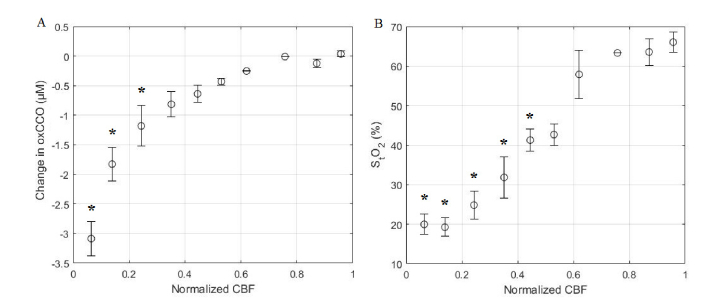
Correlation plots of a) oxCCO vs CBF, and b) S_t_O_2_ vs CBF; * indicates a significant change in either oxCCO or S_t_O_2_ from baseline (p<0.05). Error bars represent the standard error of the mean. CBF intervals with mean values of 0.62 and 0.76 show only data from two and one animals, respectively.

To further investigate the temporal relationship between CBF and metabolism during HI, oxCCO and S_t_O_2_ were separately correlated to incremental changes in CBF as described in section 2.5. Data presented in Fig. 8(A)

and Fig. 8(B) show oxCCO and S_t_O_2_, respectively, averaged across all animals and plotted against averaged changes in CBF normalized to baseline values. Due to the rapid and large initial reduction in CBF caused by carotid occlusion, the initial drop in CBF changes was greater than 40% in some experiments. Consequently, CBF intervals with mean values of 0.62 and 0.76 show only data from two and one animals, respectively.

## 4. Discussion

In this article, we report on the development of a hybrid diffuse correlation spectroscopy (DCS) and broadband near-infrared spectroscopy (B-NIRS) instrument capable of simultaneous monitoring of cerebral blood flow (CBF) and changes in the oxidation state of cytochrome c oxidase (oxCCO). This was achieved using a simple shutter-based multiplexing method that allowed acquisition of DCS and B-NIRS measurements in quick succession, thereby avoiding crosstalk between the two subsystems.

Although the importance of monitoring both CBF and metabolism has been long recognized, S_t_O_2_ is still the most widely used marker of cerebral health in the clinic, despite its limited sensitivity to brain injury [8]. The lower sensitivity of S_t_O_2_ is believed to be related, in part, to the inability to separate blood flow and metabolic effects [8,33]. Hemoglobin difference (Δ[HbDiff] = Δ[HbO_2_] - Δ[Hb]) has been used as a surrogate marker of CBF, but its relationship to flow also depends on oxygen metabolism [33]. Recent advancements have enabled direct CBF monitoring by DCS; however, monitoring CBF alone may not be sufficient to capture the interplay between substrate delivery and demand. To assess clinically significant changes in CBF, which could impact tissue viability, measures of metabolism have been recently investigated. Conversely, monitoring CBF could help determine if metabolic reductions are due to limited oxygen delivery as opposed to changes in demand.

The multiplexing approach presented here was necessary to avoid crosstalk between the B-NIRS and DCS techniques as the DCS laser would saturate the B-NIRS detector if they are operated simultaneously. As well, when the broadband source is used concurrently with DCS measurements, the autocorrelation curves are altered, particularly at short distances between the B-NIRS emission probe and DCS detection fiber. Even when a single-mode DCS detection fiber was used, to maximize the dynamic range of the correlation factor, we found that with a 10 mm distance between the B-NIRS source and the DCS detection, incoherent light excited additional modes in the DCS fiber. This manifested as a much lower β value in the autocorrelation curves (Fig. 2) and resulted in a significantly different diffusion coefficient (p < 0.01), warranting the use of shutters. Figure 3(A) shows that increasing the B-NIRS light intensity decreases the correlation factor (y-intercept). However, the values of diffusion coefficients obtained by fitting the measured autocorrelation curves in Fig. 3(A) with the solution to the diffusion equation for a semi-infinite homogeneous medium [34] did not significantly differ, as shown in Fig. 3(B). This shows that at larger distances, the B-NIRS light affects the correlation factors of the autocorrelation curves but has no significant effect on the diffusion coefficient.

The SNR can be further increased by reducing the sampling rate; lengthening the shutter cycle times allows for measures to be averaged over more acquisitions, but this would increase the time between successive measurements. In its current configuration, the hybrid B-NIRS/DCS device provided S_t_O_2_, CBF, and oxCCO measurements every 14 s. This sampling rate could likely be increased to every 3-5 s using new technologies such as the DCS software correlator [35]. However, the speed of the current system was sufficient to capture the dynamics of the CBF and oxCCO responses to HI, and therefore will likely be sufficient for cerebral monitoring in the NICU.

S_t_O_2_ was monitored by a combination of a spectral derivative fitting approach to determine baseline S_t_O_2_ and a faster linear algorithm based on the Beer-Lambert law to track subsequent changes in HbO_2_ and Hb concentrations. The reproducibility of the baseline optical properties derived from the former was tested by conducting an error analysis involving Monte Carlo simulations. Figure 4 and Table 2 demonstrate the ability of the fitting technique to extract parameter estimates with good precision and accuracy. These estimates were found to be insensitive to the boundary conditions and initial values used by the fitting routine (Table 1). Furthermore, the average baseline S_t_O_2_ (64 ± 13%) was in good agreement with a previous study (S_t_O_2_ = 69 ± 2%) involving the same animal model but using time-resolved NIRS to measure the optical properties [12]. Further validation studies are on-going to assess the accuracy of this technique over a wide range of S_t_O_2_ [36]; however, it is important to realize that the computational efficiency of the UCLn algorithm is extremely valuable for real-time monitoring in clinical applications.

Source-detector distances of 3 cm for B-NIRS and 2 cm for DCS were chosen to probe the piglet brain because it has been shown that in reflectance geometry, the penetration depth is approximately the square root of the source-detector distance [37]. Considering that the thickness of the extra-cerebral layers in newborn piglets is 2-3 mm [23], the brain can be reliably probed using a source-detector distance of 2 cm or greater. Note that this approach has been previously validated against CT perfusion measurements in a neonatal piglet model [23]. In addition, the probe geometry depicted in Fig. 1 was designed to ensure that DCS and B-NIRS probed overlapping brain regions. It is important to note that the HI model causes global changes in cerebral blood flow and metabolism. Therefore, it is expected that small differences in the tissue volumes interrogated by the two instruments will not alter the relationship between the measured hemodynamic and metabolic changes.

There have been a number of previous studies combining B-NIRS and DCS [38–40], in particularly Lee et al. recently presented a compact device for dual monitoring of tissue flaps. However, the greater source-detector distances required for brain monitoring limits the applicability of many of these approaches. Furthermore, a key novelty of our method is the ability to continuously monitor S_t_O_2_, absolute blood flow, and oxCCO, which has not been previously reported. In terms of brain monitoring, a similar concept combining frequency-domain NIRS (FD-NIRS) and DCS [41] was recently reported. This device is capable of simultaneous monitoring of S_t_O_2_, CBF, and CMRO_2_, and has been used to investigate neonatal hemodynamics. Instead of a 785-nm laser, a DCS laser emitting at 850 nm was used along with optical filters to prevent crosstalk between the two systems. An 850-nm laser is outside of the spectral range used for broadband analysis of hemoglobin concentration (680 to 845 nm), but more critically it is within the range of the CCO feature (780 to 900 nm). Although there are long coherence lasers operating at wavelengths lower than 680 nm, it is noteworthy that they have significantly lower power outputs. As well, above 900 nm, SPCM detectors commonly used in DCS systems have lower quantum efficiencies; both factors would reduce SNR. These limitations of current technology restrict the feasibility of using optical filters for concurrent monitoring of CBF and oxCCO.

The HI piglet model produced rapid and dynamic changes in S_t_O_2_, CBF, and oxCCO. More importantly, the experimental results clearly demonstrated the unique individual responses in oxygenation, flow, and metabolism (Fig. 7 and 8). These differences warrant the inclusion of all three measures in order to understand the broader physiological impact of HI. That is, S_t_O_2_ and hemoglobin concentrations allow for monitoring of hypoxia, CBF dynamics indicate ischemia, and changes in oxCCO relate to metabolic stress. Together, these different perspectives describe the hemodynamic and metabolic environment that surrounds and precedes brain injury.

In the 6 animals with HI injury, the decrease of oxCCO was delayed in comparison to CBF and continued to decline even after CBF and S_t_O_2_ reached their nadirs. A similar biphasic relationship between oxCCO and HbDiff was found in piglets experiencing anoxia [42]. Group averages of time courses are not shown due to the temporal variability between clamping carotid arteries and reducing oxygen content. The timing of the latter varied because it was only executed once occluders were shown to produce a noticeable decrease in CBF. Nevertheless, time courses across all experiments showed the direct effect of decreased CBF on S_t_O_2_ and a delayed oxCCO response. Reduced blood flow is likely met by increased oxygen extraction in order to maintain oxidative metabolism, which is consistent with previous reports between CBF and CMRO_2_ in piglets [33] and infants [9]. This compensatory mechanism may explain the delayed oxCCO response. However, the relationship between oxidative metabolism and oxCCO is complex. Further investigation into the interrelationship between these values and their physiological significance in predicting brain injury will be the subject of future investigations.

## 5. Conclusion

This article reports on the development of a novel optical system that combines DCS and B-NIRS to enable continuous monitoring of *absolute* cerebral blood flow and changes in the oxidation state of cytochrome c oxidase − two sensitive biomarkers of brain health. The combination of the two subsystems was achieved through the use of a simple multiplexing approach based on electromechanical shutters and can be easily implemented elsewhere. In addition, the ability to simultaneously monitor CBF and oxCCO has led to a key finding; that is, the response of cerebral oxygen metabolism is delayed in hypoxia-ischemia while the CBF response is instantaneous. We anticipate that real-time monitoring of absolute CBF and oxCCO will provide greater insights into hemodynamic events that precede brain injury and could become valuable for guiding therapeutic interventions. Furthermore, because the hybrid B-NIRS/DCS system is safe and portable, it could be easily deployed in the NICU to monitor cerebral perfusion and metabolism at the bedside in early stages following birth, with the goal of detecting significant hemodynamic events before brain injury occurs.
